# Chronic alcohol-induced neuroinflammation involves CCR2/5-dependent peripheral macrophage infiltration and microglia alterations

**DOI:** 10.1186/s12974-020-01972-5

**Published:** 2020-10-09

**Authors:** Patrick P. Lowe, Caroline Morel, Aditya Ambade, Arvin Iracheta-Vellve, Erica Kwiatkowski, Abhishek Satishchandran, Istvan Furi, Yeonhee Cho, Benedek Gyongyosi, Donna Catalano, Eric Lefebvre, Laurent Fischer, Star Seyedkazemi, Dorothy P. Schafer, Gyongyi Szabo

**Affiliations:** 1 Department of Medicine, University of Massachusetts Medical School, Worcester, MA USA; 2 Department of Medicine, Division of Gastroenterology, Beth Israel Deaconess Medical Center, Harvard Medical School, 330 Brookline Avenue, ST-214B, Boston, MA 02215 USA; 3 Allergan, South San Francisco, CA USA; 4 Department of Neurobiology, University of Massachusetts Medical School, Worcester, MA USA

**Keywords:** Macrophage, Microglia, Alcohol, Neuroinflammation, Cytokines

## Abstract

**Background:**

Chronic alcohol consumption is associated with neuroinflammation, neuronal damage, and behavioral alterations including addiction. Alcohol-induced neuroinflammation is characterized by increased expression of proinflammatory cytokines (including TNFα, IL-1β, and CCL2) and microglial activation. We hypothesized chronic alcohol consumption results in peripheral immune cell infiltration to the CNS. Since chemotaxis through the CCL2-CCR2 signaling axis is critical for macrophage recruitment peripherally and centrally, we further hypothesized that blockade of CCL2 signaling using the dual CCR2/5 inhibitor cenicriviroc (CVC) would prevent alcohol-induced CNS infiltration of peripheral macrophages and alter the neuroinflammatory state in the brain after chronic alcohol consumption.

**Methods:**

C57BL/6J female mice were fed an isocaloric or 5% (v/v) ethanol Lieber DeCarli diet for 6 weeks. Some mice received daily injections of CVC. Microglia and infiltrating macrophages were characterized and quantified by flow cytometry and visualized using CX3CR1^eGFP/+^ CCR2^RFP/+^ reporter mice. The effect of ethanol and CVC treatment on the expression of inflammatory genes was evaluated in various regions of the brain, using a Nanostring nCounter inflammation panel. Microglia activation was analyzed by immunofluorescence. CVC-treated and untreated mice were presented with the two-bottle choice test.

**Results:**

Chronic alcohol consumption induced microglia activation and peripheral macrophage infiltration in the CNS, particularly in the hippocampus. Treatment with CVC abrogated ethanol-induced recruitment of peripheral macrophages and partially reversed microglia activation. Furthermore, the expression of proinflammatory markers was upregulated by chronic alcohol consumption in various regions of the brain, including the cortex, hippocampus, and cerebellum. Inhibition of CCR2/5 decreased alcohol-mediated expression of inflammatory markers. Finally, microglia function was impaired by chronic alcohol consumption and restored by CVC treatment. CVC treatment did not change the ethanol consumption or preference of mice in the two-bottle choice test.

**Conclusions:**

Together, our data establish that chronic alcohol consumption promotes the recruitment of peripheral macrophages into the CNS and microglia alterations through the CCR2/5 axis. Therefore, further exploration of the CCR2/5 axis as a modulator of neuroinflammation may offer a potential therapeutic approach for the treatment of alcohol-associated neuroinflammation.

## Introduction

Alcohol use disorder (AUD) impacts millions of people across the world, including at least 17 million US patients [1]. Nearly 100,000 patients die each year because of AUD [1], which induces organ injury throughout the body, including in the liver and in the brain. In both humans and mice, alcohol intoxication leads to central nervous system (CNS) inflammation and neurodegeneration [2–4]. Recent studies using animal models have shown that inflammatory signaling not only contributes to neurodegeneration but also to alcohol addiction [5–7], making targeting of neuroinflammation a critical approach in the treatment of AUD.

Both infiltrating macrophages (IMs) and microglia become activated in response to tissue damage and can release proinflammatory cytokines, which may contribute to neuroinflammation and blood-brain barrier breakdown [8, 9]. Further, microglia, a resident macrophage of the CNS, become activated in the brain after alcohol use [8, 9]. In addition to microglia, peripheral macrophages can be recruited into the CNS under pathologic conditions and may serve to amplify ongoing neuroinflammation [10]. Recent evidence suggests that breakdown of the blood-brain barrier occurs in postmortem tissue of AUD patients, and knockout of Toll-like receptor 4 (TLR4), an important innate immune signaling receptor expressed by microglia and peripheral macrophages, protects from the ensuing alcohol-induced neuroinflammation [11]. While microglial activation has been studied in alcohol-induced neuroinflammation, the potential infiltration of peripherally recruited macrophages is yet to be evaluated.

Peripheral macrophage chemotaxis through the receptor-ligand CCR2-CCL2 signaling axis is important in macrophage recruitment to the CNS. Recent studies have found an increase in the expression of the CCR2 ligand, CCL2 (also called monocyte chemoattractant protein 1; MCP1), in the brain after chronic alcohol consumption [8, 12, 13]. Cenicriviroc (CVC) is a small molecule inhibitor recently developed to block signaling through CCR2, as well as the chemokine receptor CCR5 that recognizes CCL5 and is involved in the recruitment of monocytes and T cells [14]. CVC is currently under investigation in a phase 3 trial to treat liver fibrosis in non-alcoholic steatohepatitis (NCT03028740), a disease that involves significant inflammation [15]. CVC has been shown previously to inhibit macrophage recruitment and activation in liver disease [16].

We hypothesized that, because the chemokine CCL2 is highly expressed in human and mouse brains after chronic alcohol use, chronic ethanol promotes infiltration of peripheral IM recruitment in the brain, those IMs contribute to ethanol-induced neuroinflammation, and treatment with CVC to inhibit infiltration of peripheral macrophages will protect from proinflammatory signaling in the CNS. Here, we present evidence of region-specific peripheral macrophage recruitment, associated with cytokine expression and microglial activation after exposure to a model of chronic alcohol in mice. Treatment with CVC reduced the number of IMs in the CNS, reduced cytokine expression, and corrected microglial morphology.

These data provide important insights into the role of IMs in alcohol-induced neuroinflammation and offer a novel target for therapeutic treatment in this preclinical model of alcohol use disorder.

## Methods

### Mice

The study protocol was approved by the Institutional Animal Use and Care Committee of the University of Massachusetts Medical School. All the methods were carried out in accordance with the approved guidelines. CX3CR1^eGFP/eGFP^ mice were provided by Dr. Dorothy Schafer from the University of Massachusetts Department of Neurobiology. CCR2^RFP/RFP^ mice were purchased from the Jackson Labs. CX3CR1^eGFP/wt^ CCR2^RFP/wt^ mice were bred in-house. Wild-type mice were purchased from the Jackson Labs and allowed to acclimate to our animal medicine facility for at least 1 week prior to experimental use. Eight-week-old female mice were used for alcohol feeding experiments. Eight-week-old C57BL/6J mice were divided into alcohol- and pair-fed (control) groups as well as two CVC alcohol-fed groups. Alcohol-fed mice received 5% (v/v) alcohol in Lieber-DeCarli liquid diet ad libitum as previously described [17]. Pair-fed animals received a calorie-matched liquid diet. Mice were provided continuous access to alcohol or calorie-matched liquid diet until the time of anesthesia and were immediately transcardially perfused once appropriate sedation was achieved.

### CVC administration

Some alcohol-fed mice received daily subcutaneous injections of 15 mg/kg body weight Cenicriviroc (CVC; provided by Tobira Therapeutics, Delaware, USA) for 6 weeks (“prevention” cohort) while others received the same dose daily for 3 weeks (beginning after 3 weeks of alcohol feeding and continued to week 6 of alcohol feeding; “treatment” cohort). Ten percent hydroxypropyl-β-cyclodextrin (Sigma, St. Louis, MO USA), 5% Kolliphor (solutol) HS15 (Sigma), and 85% sterile water were used as a vehicle to dissolve CVC into a solution. Pair-fed mice and alcohol-fed mice received daily injections of the vehicle without CVC for 6 weeks.

### Brain immune cell isolation

Mice were anesthetized and transcardially perfused with PBS/heparin (Hospira, Lake Forest, IL, USA) to clear blood cells from the vasculature. The brains were dissected out and meninges removed. The tissue was then homogenized with a Tenbroek homogenizer (Corning, Corning, NY, USA), and the cell suspension was then passed through a 70-μm filter. This single-cell suspension was then applied to a 70/50/35% Percoll Plus (GE Healthcare, Pittsburgh, PA, USA) density gradient and spun at 2000*g* for 20–30 min. Microglia and macrophages collected at the interphase were then washed with PBS before proceeding.

### Flow cytometry

Isolated immune cells were stained with the Live/Dead Fixable Blue Dead Cell Stain Kit from Life Technologies (Grand Island, NY, USA) to exclude dead cells. Anti-mouse CD16/CD32 mAb from BD Biosciences (San Jose, CA, USA) was used to block non-specific Fc receptor binding and incubated for 20 min at 4 °C. Cells were immunostained for 30 min at 4 °C with surface antibodies (Table 1), fixed and permeabilized with BD Biosciences Cytofix Cytoperm Plus according to the manufacturer’s protocol, and stained for intracellular CD68 for 30 min at 4 °C (Table 1). Data were acquired on a BD Biosciences LSR II instrument and analyzed using the FlowJo v10.1 software (Ashland, OR, USA).

**Table 1:** Flow cytometry and immunofluorescence antibodies

**Antigen**	**Color**	**Clone**	**Company**	**Cat. no.**	
CD11b	APC	M1/70	Biolegend	101212	
CD45	PE/Cy7	30-F11	eBioscience	25-0451-82	
CD68	PerCP/Cy5.5	FA-11	Biolegend	137010	
CD86	FITC				
MHC-II	APC/Cy7	M5/114.15.2	Biolegend	107628	
CD206	PE	C068C2	Biolegend	141706	
CD163 (goat host)	n/a	K-18	Santa Cruz	sc-18796	
Goat IgG	Qdot 525	n/a	Invitrogen	Q22072	
**Primary antigen**	**Primary dilution**	**Company**	**Cat. no.**	**Secondary and fluor**	**Secondary dilution**
IBA1	1:500	Wako		Anti-Rb-488	1:500
CD68	1:500	AbD Serotec	MCA1957	Anti-Rt-647	1:500

Secondary antibodies from Life Technologies

### Tissue preparation and confocal microscopy

Mice were anesthetized and transcardially perfused with PBS/heparin (Hospira, Lake Forest, IL, USA) to clear blood cells from the vasculature. The brains were dissected out and meninges removed and fixed in 4% paraformaldehyde (Boston Bioproducts, Ashland, MA, USA) for 3–4 h at room temperature then cryopreserved in 30% sucrose overnight at 4 °C. Tissue was then placed in OCT Compound (Tissue Tek, Torrance, CA, USA) and frozen at − 20 °C. Twelve to 14 μm sagittal sections were cut using a Leica LM3050S cryostat (Buffalo Grove, IL, USA). The tissue sections were washed with PBS, blocked in 1% BSA (Fisher BioReagents, Fair Lawn, NJ, USA) and 1% normal goat serum (Invitrogen, Carlsbad, CA, USA) with 0.3% Triton X-100 (Sigma) at room temperature for 2 h, stained overnight with the appropriate primary antibodies (Table 1) at 4 °C, washed, stained with appropriate secondary antibodies (Table 1) for 1 h at room temperature, and mounted with Prolong Gold Antifade Reagent (Invitrogen). Images were acquired using either an LSM 700 scanning confocal microscope with Zeiss Imager.Z2 or a Zeiss Observer.Z1 confocal microscope equipped with the Zen Blue acquisition software (Zeiss, Oberkochen, Germany).

### Analysis of microglial immunofluorescence

Microglia were identified within the hippocampus, and soma area and the perimeter length of microglial extensions were measured using Fiji v1.0. Somas were measured by drawing a circumferential outline along the edge of the cell body to the base of any cellular extensions. The perimeter length was measured by summing the lengths of all cellular extensions.

### RNA extraction and qPCR

Brain tissue was dissected from mice perfused with PBS/heparin, and the hippocampus, cerebellum, and cortex tissue were stored in RNA later. The cortex tissue was defined as the superficial tissue (from the surface to approximately 0.5 mm deep) dissected approximately 2 mm anterior, 2 mm posterior, and 3 mm lateral with respect to the bregma, a region that encompasses the motor and somatosensory cortex. RNA was extracted using the miRNeasy Kit (Qiagen, Germantown, MD, USA) with on-column DNAse digestion (Zymo Research, Irvine, CA). Concentration was determined using a Nanodrop 2000 (Thermo Scientific, Waltham, MA, USA), and 1 μg RNA was used for cDNA reverse transcription (BioRad, Hercules, CA, USA). Quantitative real-time polymerase chain reaction (qPCR) was completed using SYBR Green polymerase (BioRad) and expression measured on a BioRad CFX96 Real Time System. qPCR primers are listed in Table 2, and the expression was quantified by using the 2^−ΔΔCt^ method.

**Table 2:** Real-time PCR primers

	Forward	Reverse
**Nanostring hits**		
*C1qa*	CAAGGACTGAAGGGCGTGAA	GGGGCTGGTCCCTGATATTG
*C1qb*	GGTGCCAACAGCATCTTCAC	TTTGACCCCGTGATTACGCA
*Daxx*	CTATAGGCCAGGCGTTGACC	GTTCGATTTTCCCGAAGGCG
*Gnas*	ATGGGTTTAACGGAGAGGGC	ACCATCGCTGTTGCTCCTTG
*Hmgn1*	CTCCTCGGTGACAGATCCGA	AACCTTCCTCTTGGGCATCG
*Hspb1*	CTGGCAAGCACGAAGAAAGG	GCACCGAGAGATGTAGCCAT
*Il-23*	TGGTTGTGACCCACAAGGAC	CAGACCTTGGCGGATCCTTT
*Map3k9*	TGGGCAGAAAGAGCTCACAT	ACATCATCTGCCTCTTACCCTTC
*Mapk1*	CAGTTTGTCCCCTTCCATTGAT	ACTCCCACAATGCACACGAC
*Mef2a*	AGCACTTTGAAAGGAAGAGTCCA	AGCTCCCCCACTGCACATTA
*Myd88*	AGGCATCACCACCCTTGATG	CGAAAAGTTCCGGCGTTTGT
*Plcb1*	AGATCCTCGATGAGAAGCCC	CTTCCGACAAGACTGAGGAGG
*Tgfβ1*	GTCACTGGAGTTGTACGGCA	AGCCCTGTATTCCGTCTCCT
*Itgb2*	TTCCTGGTGCCAGAAGCTGAA	CCCCGTTGGTCGAACTCAG
*Ccl11*	TGCAGGCAGTTTTCTCTGGA	AGGCTCTCCCGACTAGCTTT
**Proinflammatory**		
*Tnfα*	GAAGTTCCCAAATGGCCTCC	GTGAGGGTCTGGGCCATAGA
*Tlr4*	TCAGAACTTCAGTGGCTGGA	AGAGGTGGTGTAAGCCATGC
*Cox2*	AACCGAGTCGTTCTGCCAAT	CTAGGGAGGGGACTGCTCAT
*Ym1*	CAGAAGCTCTCCAGAAGCAAT	TGCCAGACCTGTGACAAGAAT
*Il-1β*	TCTTTGAAGTTGACGGACCC	TGAGTGATACTGCCTGCCTG
* Il-17*	CAGGGAGAGCTTCATCTGTGT	GCTGAGCTTTGAGGGATGAT
**CCR2/5 network**		
*Ccl2*	CCACAACCACCTCAAGCACT	AGGCATCACAGTCCGAGTCA
*Ccl3*	ATATGGAGCTGACACCCCGA	TCAACGATGAATTGGCGTGG
*Ccr2*	GTGTACATAGCAACAAGCCTCAAAG	CCCCCACATAGGGATCATGA
*Ccr5*	TGGGGTGGAGGAGCAGGGAG	TAGGCCACAGCATCGGCCCT
**Housekeeping**		
*Gapdh*	GGCAAATTCAACGGCACAGT	GATGGGCTTCCCGTTGATGA
*18S rRNA*	GTAACCCGTTGAACCCCATT	CCATCCAATCGGTAGTAGCG

### Nanostring gene expression analysis

RNA was extracted as above. RNA and Nanostring reactions were prepared according to the manufacturer’s recommendation for the Mouse nCounter Inflammation Panel (Nanostring Technologies, Seattle, WA, USA). Data were analyzed using the nSolver Analysis Software 3.0 (Nanostring Technologies).

### Two-bottle choice test

A continuous access (24 h access) two-bottle choice between alcohol and drinking water was used to measure alcohol consumption [18]. Briefly, mice were housed singly and were provided two water-only graduated glass feeders [19] for a 1-day acclimation period along with a chow diet which was provided ad libitum throughout the experiment. Thereafter, one feeder was filled with alcohol and the other water. Mice consecutively received 4 days each of 3, 6, 9, and 12% (v/v) alcohol in water. Alcohol was made fresh daily, and the alcohol feeder location was changed to control for side preference bias. Consumption volumes were measured daily and a control cage without mice was included on each mouse rack to correct for spillage of liquid throughout the 24-h period. In the event of leakage from a feeder or if cage bedding was stuffed into the feeder by a mouse, data from that cage for that day was excluded from the analysis. Alcohol consumption and preference were calculated as previously described [18]. Some wild-type female C57BL/6J were treated with daily i.p. injections of 15 mg/kg body weight CVC or with an equal volume of the vehicle.

### Statistical analysis

Statistical significance was determined using Student *t* test or ordinary one-way ANOVA with Tukey’s multiple comparison post-test to compare the means of multiple groups. Data are shown as mean ± SEM and were considered statistically significant at *p* < 0.05. For the two-bottle choice test analysis, we used the two-way analysis of variance (ANOVA) with post hoc Bonferroni with GraphPad Prism 7.0c (GraphPad Software Inc., La Jolla, CA, USA).

## Results

### Alcohol induces recruitment of peripheral macrophages into the CNS

**Fig. 1:**
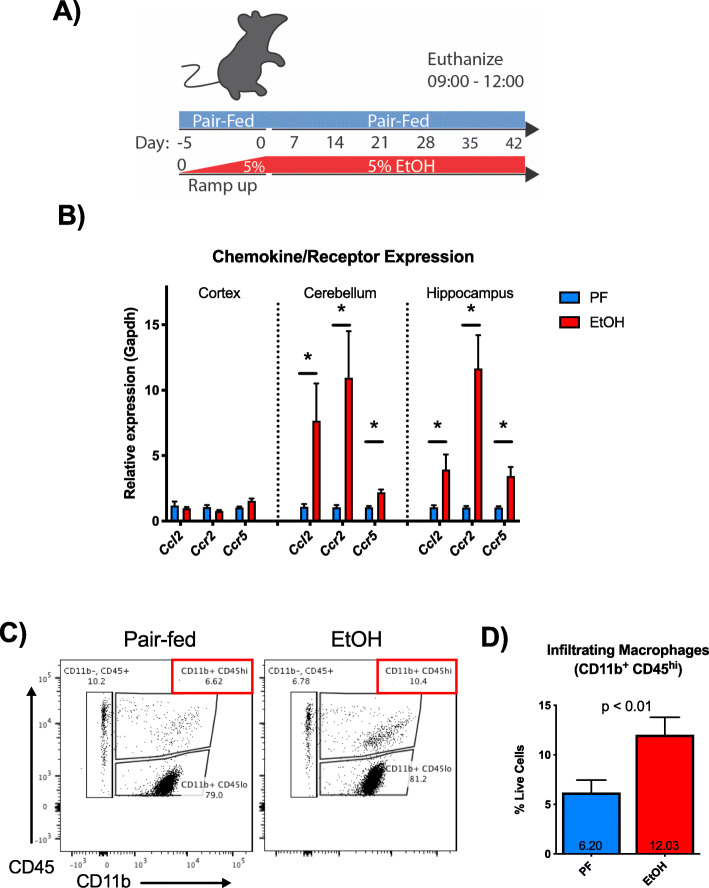
Alcohol induces infiltration of peripheral macrophages into the CNS. **a** Alcohol-fed mice received 5% (v/v) alcohol in Lieber-DeCarli liquid diet ad libitum (EtOH) for 6 weeks while pair-fed animals received a calorie-matched liquid diet (PF). **b** mRNA expression of *Ccl2*, *Ccr2*, and *Ccr5* was measured from the hippocampus of PF and EtOH mice. **c** Brain immune cells from wild-type mice fed chronic alcohol (EtOH) or a pair-fed (PF) control diet were isolated and quantified by flow cytometry gating for live, single cells. Microglia (CD11b^+^ CD45^lo^) were differentiated from peripheral macrophages (CD11b^+^ CD45^hi^) based on surface marker staining and infiltrating macrophages in pair- vs alcohol-fed mice. **d** Quantification of microglia (CD11b^+^ CD45^lo^) and peripheral macrophages (CD11b^+^ CD45^hi^) in PF and EtOH mice. Data are mean ± SEM, *n* = 4–7 mice/group. *p < 0.05 by Student’s *t* test

In order to investigate the role of peripheral infiltrating macrophages (IMs) in chronic alcohol, we used a common model [20] of chronic alcohol consumption in mice (Fig. 1a). We measured the mRNA expression of the chemokine receptors *Ccr2* and *Ccr5* and the macrophage chemokine *Ccl2*, a ligand for CCR2, in the CNS. Alcohol significantly induced *Ccl2*, *Ccr2*, and *Ccr5* expression compared with pair-fed controls in both the cerebellum and hippocampus (*p* < 0.05), but not in the cortex (Fig. 1b). To test if this upregulation of monocyte chemokines and receptors is associated with the infiltration of macrophages, we next isolated CNS immune cells from the total brain and examined the cells using flow cytometry. Peripheral IMs were defined by flow cytometry as CD11b^+^CD45^hi^ cells to differentiate from CD11b^+^CD45^lo^ microglia (Fig. 1c). CD11b^+^CD45^hi^ IMs were significantly more abundant in alcohol-fed mice compared to pair-fed mice (6.2 ± 0.52% vs 12.03 ± 1.64%, *p* < 0.01) (Fig. 1d). These data suggested that peripheral immune cells indeed infiltrate into the CNS after alcohol feeding and, based on the differential expression of chemokine receptor and ligand, that they may be recruited to particular brain regions.

**Fig. 2:**
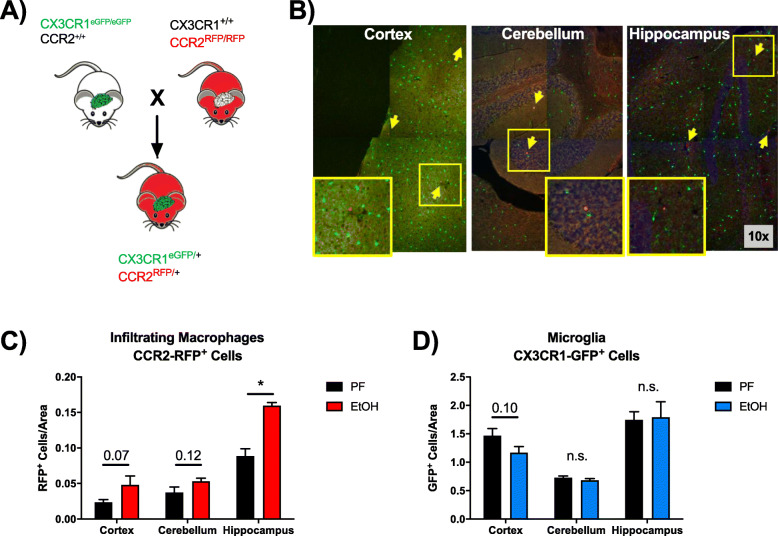
Alcohol-induced peripheral macrophages differentially infiltrate the CNS. **a** CX3CR1^eGFP/+^ CCR2^RFP/+^ mice were generated by crossing CX3CR1^eGFP/eGFP^ CCR2^+/+^ and CX3CR1^+/+^ CCR2^RFP/RFP^ mice to allow visualization of resident microglia (GFP^+^; green) and brain infiltrating macrophages (RFP^+^; red) in the cortex, cerebellum, and hippocampus of mice fed chronic alcohol or a calorie-matched diet. **b** Acquired × 10 images of ethanol-fed CX3CR1^eGFP/+^ CCR2^RFP/+^ mice were stitched together to provide the larger representative images shown. **c**, **d** Quantification of resident CX3CR1-eGFP^+^ microglia and CCR2-RFP^+^ brain infiltrating macrophages in the cortex, cerebellum, and hippocampus. Data are mean ± SEM, *n* = 4–7 mice/group. *p < 0.05; n.s., not significant by Student’s *t* test

To better understand the regional distribution of peripheral macrophages identified using flow cytometry from the total brain, we crossed CX3CR1^eGFP/eGFP^ and CCR2^RFP/RFP^ mice to yield CX3CR1^eGFP/+^ CCR2^RFP/+^ mice (Fig. 2a), allowing for the differentiation of microglia (CX3CR1^eGFP^) and IMs (CCR2^RFP^). CCR2^RFP^ macrophages were identifiable in the cortex, cerebellum, and the hippocampus of ethanol-fed mice (Fig. 2b). Although there was a general trend toward an increase in the number of peripheral CCR2^RFP+^ IMs after chronic alcohol in all three brain regions, IMs were twice as numerous in the hippocampus in the ethanol-fed mice (Fig. 2c). Interestingly, there was no change in the number of CX3CR1^eGFP/+^ microglia in any of the brain regions (Fig. 2d).

### Inhibition of CCR2/5 signaling with cenicriviroc reduces CNS macrophage infiltration without modulating their activation state

**Fig. 3:**
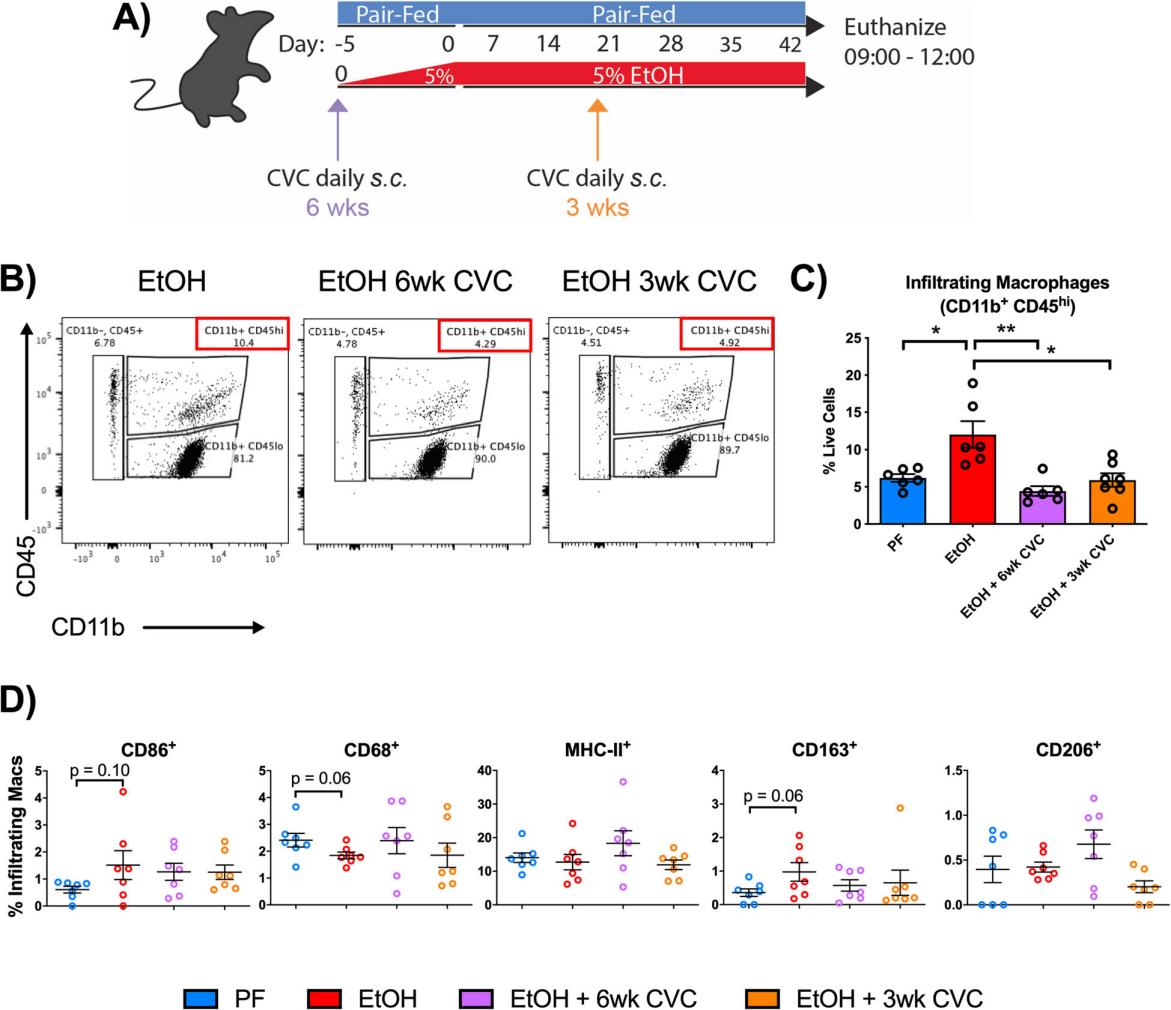
Inhibition of CCR2/5 signaling reduces CNS macrophage infiltration without altering activation marker expression of infiltrating macrophages. **a** Mice received a pair-fed diet (PF) or chronic alcohol (EtOH), and some alcohol-fed mice received 6 weeks of daily preventive subcutaneous CVC injection (EtOH + 6wk CVC) or 3 weeks of daily CVC treatment (EtOH + 3wk CVC). PF and EtOH mice received daily vehicle control injections. **b** Representative flow cytometry plots of peripheral macrophages (CD11b^+^ CD45^hi^) in EtOH, EtOH + 6wk CVC and EtOH + 3wk CVC-treated mice. **c** Quantification of infiltrating brain macrophages (CD11b^+^ CD45^hi^) in pair-fed, alcohol-fed, and treatment groups. **d** Expression of various activation markers were measured by flow cytometry in CD11b^+^ CD45^hi^ infiltrating macrophages including CD86, CD68, MHC-II, CD163, and CD206. Data are mean ± SEM, *n* = 6–7 mice/group. *p < 0.05 by one-way ANOVA

To investigate the role of IMs that infiltrate into the CNS after chronic alcohol, we used a small molecule inhibitor, cenicriviroc (CVC), that blocks the chemokine receptors CCR2 and CCR5, receptors expressed on monocytes and macrophages, among other immune cells [16, 21]. Some mice were fed chronic alcohol and were treated daily with CVC throughout alcohol exposure for a total of 6 weeks of treatment (6wk CVC), while other mice fed chronic alcohol were only treated with CVC for the final 3 weeks (3wk CVC), mimicking a clinical treatment paradigm where treatment often begins only after exposure has occurred (Fig. 3a). Pair-fed and alcohol-fed mice received daily vehicle control injections. Alcohol significantly increased CD11b^+^CD45^hi^ IMs compared to pair-fed controls (Fig. 3b). Both 6wk CVC and 3wk CVC treatment paradigms abrogated the alcohol-induced infiltration of CD11b^+^CD45^hi^ IMs, significantly reducing IMs to the level of pair-fed mice (Fig. 3b, c).

Previously, we and others have shown that proinflammatory cytokine expression is increased in the brain after chronic alcohol exposure [12, 13, 22]. Because of the proinflammatory environment in the CNS induced by alcohol consumption, we hypothesized that macrophages may have altered the expression of surface activation markers. Using flow cytometry of isolated CNS immune cells from the total brain, we observed that alcohol modestly altered the expression of multiple investigated activation markers of the CD11b^+^CD45^hi^ macrophages, including CD86, CD68, MHC-II, CD163, and CD206 (Fig. 3d). The expression of these markers was not significantly changed on CNS IMs, although an increasing trend was observed for CD86 and a decreasing trend for CD163. CVC treatment did not significantly alter the expression of these markers on CD11b^+^CD45^hi^ macrophages (Fig. 3d), suggesting that the activation state of IMs in the CNS in alcohol-fed mice is independent of CCR2/5 signaling.

### Chronic alcohol induces inflammatory protein and gene expression changes

**Fig. 4:**
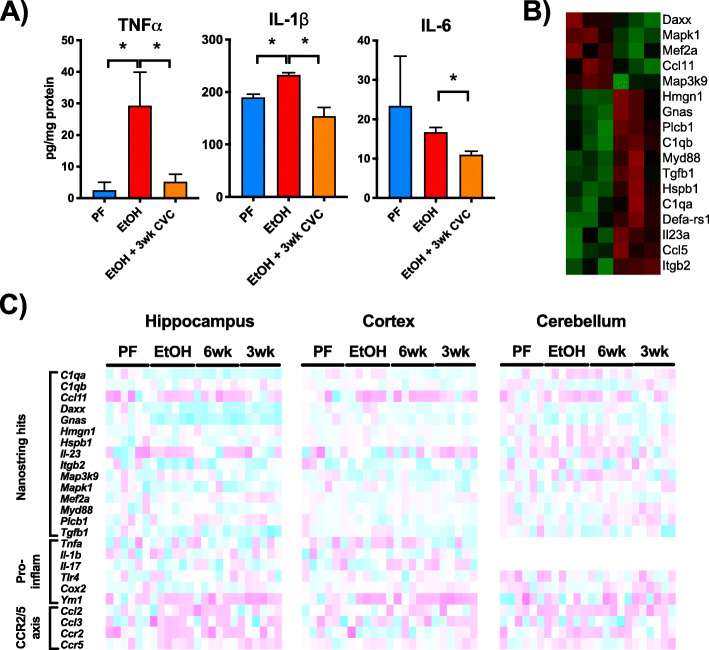
Chronic alcohol induces inflammatory gene expression changes in multiple brain regions. **a** Proinflammatory cytokine proteins TNFα, IL-1β, and IL-6 were measured from the hippocampus of pair- (PF) and alcohol-fed (EtOH) as well as alcohol-fed mice treated with CVC for 3 weeks (EtOH +3wk CVC) by ELISA. **b** Inflammatory gene expression of pair- and alcohol-fed mice was analyzed using the Nanostring nCounter Immunology Panel and revealed 17 genes significantly altered in the cerebellum (green, expression increased; red, expression decreased). **c** Gene expression changes of genes found by Nanostring to be altered as well as some common inflammatory markers, and CCR2/5 axis genes were measured in the hippocampus, cortex, and cerebellum by qPCR in a larger cohort of pair- and alcohol-fed mice as well as mice treated with 3 or 6 weeks CVC (purple, > 3-fold increase in expression; cyan > 3-fold decrease in the expression compared to PF; complete gene expression profile data found in Tables 3, 4, and 5). Data are mean ± SEM, *n* = 4–6 mice/group. *p < 0.05 by one-way ANOVA

We measured protein levels of proinflammatory cytokines including TNFα, IL-1β, and IL-6 in the hippocampus, where we had observed a significant increase in CCR2^+^ macrophages following chronic alcohol. We found that both TNFα and IL-1β (including both cleaved and uncleaved forms) were induced by chronic alcohol and that 3-week treatment with CVC reduced these proteins as well as IL-6 in CVC-treated alcohol-fed mice (Fig. 4a).

To further probe the expression of proinflammatory cytokines and related immune genes, we used the Nanostring Inflammation Panel to screen mRNA levels of 254 inflammation-related genes. In this screen, we found 17 immune-related genes that were significantly altered in the cerebellum (Fig. 4b). We followed this screen with qPCR quantification of these gene targets as well as other selected proinflammatory targets and chemokine ligands and receptors associated with CCR2/5 signaling (Fig. 4c). Including more samples as well as samples from mice treated with CVC, we found that gene expression was significantly altered in the hippocampus, cortex, and cerebellum and that CVC treatment corrected some of these alterations (Fig. 4c). Prominently, we observed an upregulation in the CCR2/5 signaling pathway in alcohol-fed mice in the hippocampus that was significantly decreased by CVC treatment in both paradigms (Fig. 4c, Table 4). Interestingly, the most significant effect of ethanol on gene expression among all inflammatory markers measured was found in the hippocampus, where we also observed the most infiltration of CCR2^+^ peripheral macrophages. Tables 3, 4, and 5 provide the mean and standard deviation for each gene and group in the cortex, hippocampus, and cerebellum as well as *p* value comparisons between pair- and alcohol-fed mice (PF vs EtOH) as well as alcohol-fed and CVC-treated alcohol-fed mice (EtOH vs 6wk CVC; EtOH vs 3wk CVC). Significant differences are denoted with an asterisk (*) and are offset in the column (Tables 3, 4, and 5).

**Table 3:** Gene expression profile of the cortex

Cortex											
	PF		EtOH		PF vs EtOH	EtOH + 6wk CVC		EtOH vs 6 weeks	EtOH + 3wk CVC		EtOH vs 3 weeks, *p* value
	Mean	Stdev	Mean	Stdev	*p* value	Mean	Stdev	*p* value	Mean	Stdev	
**Nanostring**											
C1qa	1.021	0.242	1.472	0.519	0.089	0.751	0.185	*0.012	0.750	0.265	*0.015
C1qb	1.015	0.196	1.016	0.292	0.996	0.959	0.272	0.797	0.882	0.243	0.426
Ccl11	1.156	0.743	14.52	12.66	0.073	4.626	3.510	*0.036	3.333	3.057	0.062
Daxx	1.020	0.219	1.331	0.303	0.080	0.751	0.108	*< 0.001	0.890	0.294	*0.038
Gnas	1.017	0.218	1.167	0.279	0.342	0.734	0.068	*< 0.01	0.687	0.173	*< 0.01
Hmgn1	1.004	0.099	1.167	0.307	0.247	1.086	0.157	0.508	0.960	0.244	0.243
Hspb1	1.017	0.204	1.275	0.143	*0.042	1.034	0.238	0.124	0.937	0.237	*0.021
Il23	1.584	1.547	0.926	0.657	0.401	2.484	2.326	0.222	2.430	0.941	*0.015
Itgb2	1.103	0.498	0.833	0.447	0.394	0.384	0.203	0.123	0.726	0.621	0.771
Map3k9	1.068	0.459	0.825	0.422	0.389	0.645	0.427	0.433	0.576	0.180	0.219
Mapk1	1.075	0.499	1.258	0.269	0.482	0.820	0.397	0.108	0.843	0.267	*0.031
Mef2a	1.019	0.205	0.913	0.210	0.420	1.093	0.384	0.402	1.072	0.391	0.437
Myd88	1.017	0.200	0.968	0.244	0.720	1.096	0.263	0.596	0.931	0.108	0.750
Plcb1	1.002	0.070	0.894	0.188	0.223	0.74	0.090	0.231	0.824	0.126	0.474
Tgfβ1	1.023	0.236	1.474	0.422	0.052	0.839	0.450	*0.038	0.657	0.194	*< 0.01
**Proinflammatory**											
Tnfα	1.135	0.651	2.597	1.694	0.081	2.085	1.196	0.530	0.782	0.796	*0.043
Il1β	1.231	0.757	1.19	0.403	0.916	0.667	0.235	0.061	1.145	0.667	0.899
Il17	1.180	0.696	0.823	0.279	0.312	1.975	0.695	*0.018	1.676	1.170	0.149
Tlr4	1.023	0.236	1.209	0.492	0.430	1.024	0.409	0.678	1.013	0.309	0.439
Cox2	1.010	0.153	1.265	0.229	0.054	1.096	0.317	0.434	1.234	0.139	0.788
Ym1	1.093	0.412	22.40	13.11	*< 0.01	8.849	9.192	*0.013	8.183	7.048	*0.041
**CCR2/5 network**											
Ccl2	1.187	0.633	0.960	0.221	0.524	2.227	0.552	*< 0.01	1.684	1.458	0.364
Ccl3	1.311	0.821	2.090	1.527	0.307	1.909	1.716	0.764	1.549	1.033	0.502
Ccr2	1.065	0.402	0.764	0.165	0.200	1.214	1.718	0.623	1.256	0.564	0.134
Ccr5	1.019	0.223	1.530	0.412	*0.027	1.081	0.378	0.060	1.296	0.518	0.436

Gene expression was measured by qPCR. Data are mean or standard deviation (Stdev), *n* = 5–6 mice/group

*p < 0.05 and significant *p* values are offset from non-significant values

**Table 4:** Gene expression profile of the hippocampus

Hippocampus											
	PF		EtOH		PF vs EtOH	EtOH + 6wk CVC		EtOH vs 6 weeks	EtOH + 3wk CVC		EtOH vs 3 weeks, *p* value
	Mean	Stdev	Mean	Stdev	*p* value	Mean	Stdev	*p* value	Mean	Stdev	
**Nanostring**											
C1qa	1.059	0.351	1.049	0.411	0.966	0.851	0.265	0.344	0.670	0.235	0.078
C1qb	1.009	0.150	1.462	0.147	*< 0.001	1.782	0.459	0.134	1.563	0.265	0.431
Ccl11	1.156	0.743	14.52	12.66	0.073	4.626	3.510	0.095	3.333	3.057	0.062
Daxx	1.029	0.261	0.821	0.398	0.310	0.733	0.217	0.645	0.622	0.253	0.327
Gnas	1.017	0.208	0.585	0.306	*0.017	0.613	0.100	0.834	0.492	0.226	0.563
Hmgn1	1.017	0.213	1.482	0.333	*0.016	1.302	0.213	0.291	1.485	0.645	0.993
Hspb1	1.044	0.322	1.357	0.432	0.185	1.274	0.278	0.703	1.013	0.283	0.134
Il23	1.804	1.911	10.62	5.981	*< 0.01	3.780	4.233	*0.045	3.099	3.019	*0.020
Itgb2	1.225	0.749	0.632	0.254	0.097	1.340	0.601	*0.024	0.913	0.471	0.227
Map3k9	1.073	0.414	2.298	1.428	0.071	1.533	0.443	0.239	1.079	0.293	0.068
Mapk1	1.087	0.409	1.033	0.242	0.785	1.041	0.570	0.976	0.887	0.251	0.329
Mef2a	1.022	0.233	1.636	0.702	0.070	1.577	0.183	0.846	1.784	0.349	0.653
Myd88	1.029	0.263	1.379	0.285	0.052	1.222	0.272	0.354	1.022	0.209	*0.033
Plcb1	1.087	0.438	1.685	0.307	*0.021	1.724	0.328	0.837	1.742	0.191	0.708
Tgfβ1	1.039	0.302	0.946	0.344	0.630	0.982	0.342	0.858	0.488	0.312	*0.036
**Proinflammatory**											
Tnfα	1.246	0.651	2.206	1.911	0.291	1.490	0.665	0.407	1.383	1.214	0.394
Il1β	2.320	0.757	2.587	1.707	0.853	1.622	0.811	0.239	1.570	0.866	0.222
Il17	1.069	0.696	1.017	0.534	0.859	1.652	0.745	0.121	1.213	0.769	0.620
Tlr4	1.047	0.236	1.772	0.856	0.083	1.773	0.891	0.999	1.496	0.728	0.562
Cox2	1.007	0.153	1.011	0.168	0.970	1.275	0.231	*0.047	1.325	0.257	*0.031
Ym1	1.093	0.412	22.40	13.11	*< 0.01	8.849	9.192	0.084	8.183	7.048	*0.041
**CCR2/5 network**											
Ccl2	1.051	0.633	3.926	2.837	*0.034	3.858	2.162	0.963	1.451	1.033	0.072
Ccl3	1.301	0.821	6.713	3.069	*< 0.01	2.188	1.338	*< 0.01	2.737	2.238	*0.035
Ccr2	1.024	0.402	11.67	6.247	*0.010	3.958	3.253	*0.035	2.955	2.310	*< 0.01
Ccr5	1.024	0.223	3.433	1.685	*< 0.01	1.659	0.404	*0.031	2.267	0.733	0.151

Gene expression was measured by qPCR. Data are mean or standard deviation (Stdev), *n* = 5–6 mice/group

*p < 0.05 and significant *p* values are offset from non-significant values

**Table 5:** Gene expression profile of the cerebellum

Cerebellum											
	PF		EtOH		PF vs EtOH	EtOH + 6wk CVC		EtOH vs 6 weeks	EtOH + 3wk CVC		EtOH vs 3 weeks, *p* value
	Mean	Stdev	Mean	Stdev	*p* value	Mean	Stdev	*p* value	Mean	Stdev	
**Nanostring**											
C1qa	1.079	0.47	2.788	1.243	*0.010	1.942	0.872	0.202	1.266	0.786	*0.030
C1qb	1.012	0.161	1.017	0.397	0.976	0.773	0.275	0.244	1.165	0.264	0.466
Ccl11	1.043	0.326	37.22	36.99	0.060	16.70	26.98	0.315	6.682	12.75	0.119
Daxx	1.015	0.182	0.972	0.265	0.750	1.132	0.524	0.519	0.704	0.214	0.083
Gnas	1.014	0.198	0.713	0.401	0.130	0.842	0.269	0.528	0.611	0.160	0.577
Hmgn1	1.196	0.977	2.302	1.353	0.136	1.531	0.486	0.219	1.183	1.154	0.155
Hspb1	1.167	0.733	2.995	1.489	*0.022	1.916	0.724	0.141	1.007	0.683	*0.014
Il23	1.277	1.110	2.449	1.791	0.203	2.278	0.849	0.836	1.032	1.011	0.122
Itgb2	1.034	0.286	2.150	1.830	0.169	1.024	0.868	0.221	1.501	1.000	0.516
Map3k9	1.220	0.987	3.344	2.486	0.080	2.916	1.733	0.736	1.033	1.249	0.069
Mapk1	1.462	1.768	1.327	0.595	0.862	1.257	0.473	0.826	1.644	1.290	0.602
Mef2a	1.094	0.584	1.249	0.160	0.544	1.232	0.317	0.906	0.715	0.525	*0.039
Myd88	1.072	0.470	1.020	0.538	0.863	1.398	0.607	0.281	1.358	0.222	0.186
Plcb1	1.004	0.098	0.995	0.390	0.955	1.324	0.370	0.164	1.313	0.337	0.161
Tgfβ1	1.027	0.257	0.997	0.641	0.918	0.697	0.466	0.376	0.698	0.363	0.343
**Proinflammatory**											
Tlr4	1.151	0.368	1.813	1.143	0.207	1.164	0.450	0.225	1.406	0.710	0.477
Cox2	1.028	0.270	1.116	0.319	0.620	1.311	0.350	0.336	1.646	0.638	0.099
Ym1	2.022	2.479	3.953	4.398	0.371	3.755	6.046	0.951	3.598	2.942	0.873
**CCR2/5 network**											
Ccl2	1.087	0.471	7.654	6.998	0.068	3.831	2.432	0.235	3.102	3.328	0.181
Ccl3	1.643	1.988	4.614	4.774	0.229	1.042	1.473	0.145	1.452	1.565	0.193
Ccr2	1.047	0.384	21.35	24.06	0.096	10.48	13.49	0.368	4.777	8.151	0.234
Ccr5	1.039	0.280	2.190	0.530	*< 0.001	1.185	0.524	*< 0.01	1.819	1.235	0.514

Gene expression was measured by qPCR. Data are mean or standard deviation (Stdev), *n* = 5–6 mice/group

*p < 0.05 and significant *p* values are offset from non-significant values

### Alcohol induces morphologic changes and alters expression of activation markers in microglia that are partially restored by CCR2/5 inhibition

**Fig. 5:**
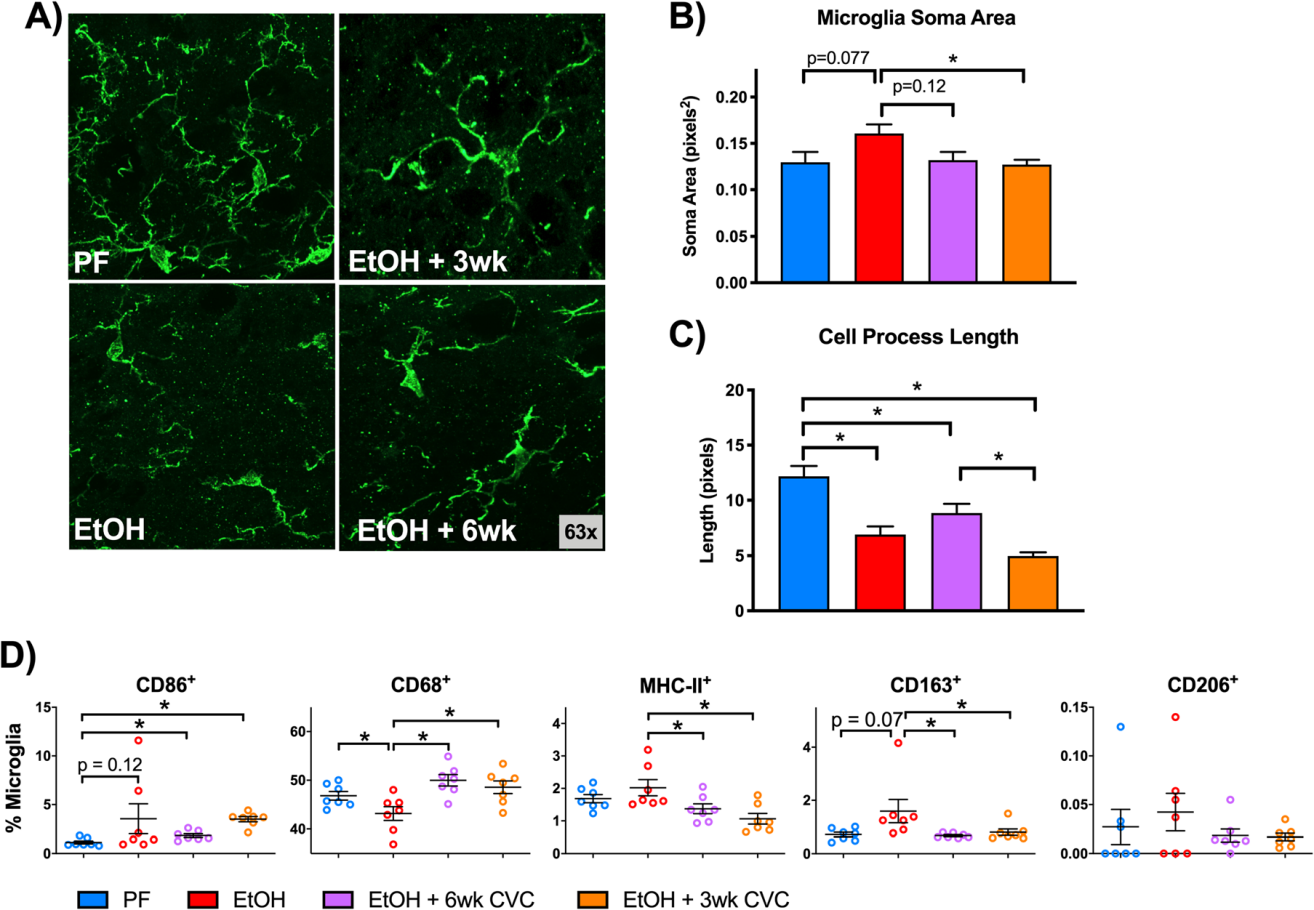
Alcohol-induced morphologic changes in microglia are partially restored by CCR2/5 inhibition. **a** Representative images of hippocampal microglia (CX3CR1-GFP^+^; green) displaying altered morphologies in pair-fed (PF) vs alcohol-fed (EtOH) mice, acquired at × 63 magnification. **b** Quantification of average hippocampal microglia soma area (pixel^2^) and **c** perimeter distance (pixel length) in mice fed control or alcohol diet or alcohol-fed mice treated with CCR2/5 inhibitor. **d** Expression of various activation markers were measured by flow cytometry in CD11b^+^ CD45^lo^ microglia including CD86, CD68, MHC-II, CD163, and CD206. Data are mean ± SEM, *n* = 3 mice/group and 3–10 sections/mouse for **a**–**c** and *n* = 6–7 mice/group for **d**. *p < 0.05 by one-way ANOVA.

Activated microglia often assume an amoeboid morphology that is characterized by shortened cell processes and an enlarged soma. We used immunofluorescent staining of the microglial marker IBA1 to investigate if chronic alcohol induces morphological changes in line with an activated cell shape (Fig. 5a). We focused on hippocampal microglia specifically because of the significant infiltration of peripheral CCR2^RFP^ macrophages to this region (Fig. 2c) suggested this as a site of active neuroinflammation. We found that chronic alcohol tended to increase the cell soma size of microglia (Fig. 5b). Inhibiting CCR2/5 signaling with CVC significantly reduced the soma in chronic alcohol-fed mice with 3 weeks of CVC treatment (Fig. 5b). Chronic alcohol significantly reduced the process length of hippocampal microglia, and CVC administration did not affect this morphology compared to chronic alcohol-fed mice (Fig. 5c). These data indicate that chronic alcohol induces a more reactive cell morphology than in pair-fed control mice and CCR2/5 inhibition modestly restores a normal cell body.

To evaluate the activation state of microglia by alcohol in the CNS, we measured the levels of CD86, CD68, MHC-II, CD163, and CD206 by flow cytometry in isolated CD11b^+^CD45^lo^ cells from the total brain. CD68 expression was downregulated in microglia following alcohol consumption, while the remainder of the activation markers were not significantly altered, although both CD86 and CD163 showed a trend toward increased expression in alcohol-fed mice. However, CVC treatment increased both CD86 and CD68 in both treatment paradigms and reduced expression of MHC-II and CD163 (Fig. 5d), suggesting that CCR2/5 signaling may contribute to microglia activation in alcohol-fed mice.

### CD68 expression is decreased by chronic alcohol

**Fig. 6:**
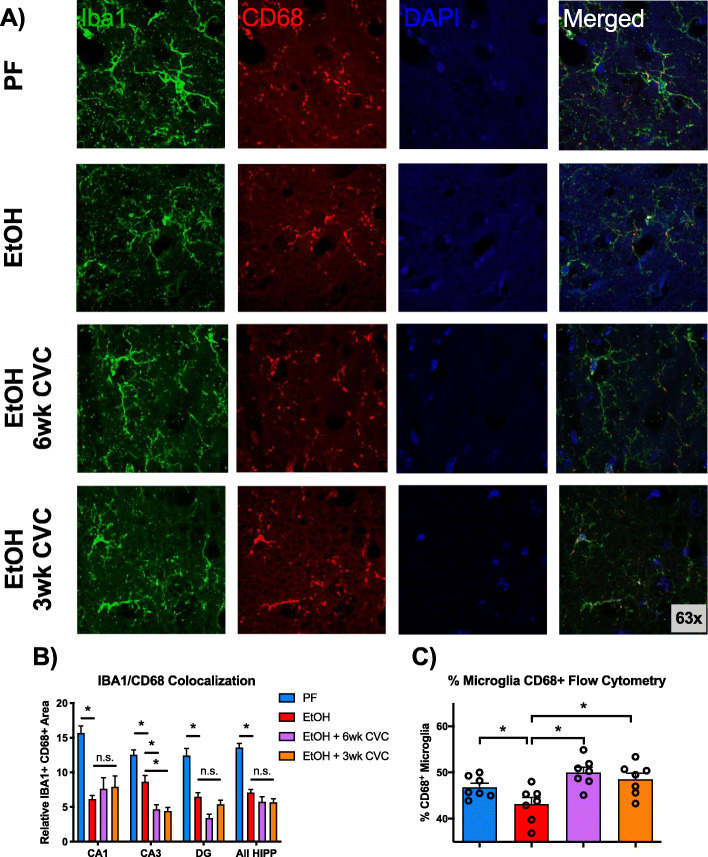
Alcohol downregulates the microglial expression of CD68. **a** Representative images of the hippocampal sections stained for Iba1 (green), CD68 (red), and DAPI (blue) from pair-fed (PF), alcohol-fed (EtOH), and CVC-treated alcohol-fed mice, acquired at × 63 magnification. **b** Quantification of Iba1 and CD68 colocalization, normalized to total Iba1^+^ area per image in the hippocampal CA1, CA3, and DG regions and throughout the HPF. **c** Flow cytometry data showing the percent of microglia (defined as CD11b^+^ CD45^lo^) that are positive for CD68 expression. Data are mean ± SEM, *n* = 3–4 mice/group with 4–5 images per region per mouse. *p < 0.05; n.s., not significant by one-way ANOVA

CD68 is a lysosomal protein that is often used as a marker of phagocytosis in macrophages and particularly microglia [23, 24]. We hypothesized that ethanol consumption not only alters microglia morphology but also its phagocytic activity in the hippocampus. Microglial CD68 levels were measured by immunofluorescence, which demonstrated a decrease in the hippocampus (including CA1, CA3, and dentate gyrus (DG) regions) of alcohol-fed compared to pair-fed mice (Fig. 6a, b). This observation was corroborated by flow cytometric analysis of isolated microglia in which we also observed a decrease in microglial CD68 positivity in alcohol-fed mice (Fig. 6c). Interestingly, treatment with CVC did not affect microglial CD68 expression (measured as colocalization of CD68 and the microglial marker IBA1) except in the CA3 region of the hippocampus where CVC decreased CD68 expression (Fig. 6b). However, flow cytometry measurements of CD68^+^ microglia revealed an effect of CVC treatment as both inhibitor treatment paradigms increased CD68 positivity (Fig. 6c). Importantly, the flow cytometry is based on microglia from the total brain, whereas the immunofluorescence measurements were performed in the hippocampus only. Taken together, these data suggest that alcohol significantly reduces microglial CD68 expression and provides evidence of alterations in the microglial phagocytic activity that may impact microglia function after chronic alcohol exposure.

### CVC administration does not affect alcohol consumption or preference

The Lieber-DeCarli alcohol delivery model relies on alcohol dissolved in a liquid diet to ensure mice receive sufficient daily alcohol for study purposes. Because the alcohol is dissolved in the animals’ only source of calories, there is limited expected variation in alcohol consumption across the groups. Indeed, treatments with CVC did not significantly alter the daily liquid diet consumption compared with vehicle-treated controls. The average daily consumption of 5% ethanol in the Lieber-DeCarli liquid diet for vehicle-treated mice was 18.73 ± 4.0 mL compared with 6wk CVC-treated mice 19.37 ± 3.6 mL (*p* = 0.37) and 3wk CVC-treated mice 19.79 ± 3.7 mL (*p* = 0.13). Therefore, to test whether CVC treatment could have an impact on alcohol consumption or preference, we used a two-bottle choice test model. Mice were treated with daily i.p. injections of CVC or with an equal volume of the vehicle and provide access to two bottles, one with water and the other with increasing concentrations of ethanol over the course of the experiment. We found that CVC treatment did not change the overall consumption of ethanol per kilogram body weight (Fig. S1A, supplementary). CVC treatment also had no significant effect on alcohol preference (Fig. S1B, supplementary) compared to vehicle-treated controls (consumption interaction *F*(3, 56) = 0.6927; *p* = 0.5603 and treatment groups *F*(1, 56) = 1.341; *p* = 0.2518; preference interaction *F*(3, 56) = 0.9346; *p* = 0.4301 and treatment groups *F*(1, 56) = 1.465; *p* = 0.2312). The total fluid was also measured as grams of ethanol and water consumed and normalized to body weight and was not statistically different between treated and untreated mice (interaction *F*(3, 56) = 0.046; *p* = 0.9869 and treatment groups *F*(1, 56) = 3.176; *p* = 0.0801) (Fig. S1C, supplementary).

## Discussion

Previous research has described neuroinflammation associated with chronic alcohol consumption. We hypothesized that alcohol could induce macrophage infiltration into the brain and that this infiltration could drive the neuroinflammation observed after chronic alcohol. We therefore tested if blockade using a CCR2/5 dual inhibitor of the chemokine network associated with macrophage chemoattraction could reduce alcohol-induced neuroinflammation. Here, we show that chronic alcohol induces region-specific infiltration of IMs into the CNS that is associated with cytokine expression and microglial activation. Inhibition of CCR2/5 signaling using the small molecule inhibitor CVC abrogated the infiltration of macrophages, reduced cytokine expression, and partially normalized microglial morphology.

Although the CNS was long considered an immune-privileged compartment, an appreciation for peripheral immune infiltration in the setting of diseases has been accepted. Only recently has infiltration of peripheral macrophages been described in the setting of alcohol-induced neuroinflammation [11, 22]. Using a different alcohol model than previously studied in mice, we confirm this observation using flow cytometry of total immune cells in the brain, detecting an increase in CD11b^+^CD45^hi^ macrophages. Peripheral macrophages have previously been distinguished from microglia (CD11b^+^CD45^lo^), and although expression of CD45 can shift during inflammation, gene expression studies suggest its expression level remains a distinguishing feature between the two cell types [25]. Additionally, we created CX3CR1^eGFP/+^ CCR2^RFP/+^ mice to allow for visualization and localization of infiltrating CCR2^+^ macrophages. While we observed a trend toward increased macrophages in the cortex and cerebellum, the largest alcohol-induced increase observed was in the hippocampus, a region of significant alcohol-related inflammation in both rodents and humans [8, 26, 27]. These data suggest that alcohol-induced macrophage infiltration into the CNS may be region-specific and possibly linked to localized neural damage and immune signaling.

Blockade of CCR2/5 signaling with CVC successfully limited the alcohol-induced infiltration of peripheral macrophages into the CNS. Previously, CVC was shown to be effective at limiting macrophage chemotaxis to the liver in a model of fibrosis [16]. While successful inhibition of peripheral immune cell infiltration is consistent with blockade of a chemokine receptor, blocking CCL2 signaling has additional advantages. In the CNS of CCL2 knockout mice, production of proinflammatory cytokines TNFα and IL-1β was significantly reduced after peripheral injection of bacterial endotoxin (lipopolysaccharide (LPS)) [28]. Interestingly, these proinflammatory cytokines were expressed even before peripheral cell infiltration occurred, suggesting that, at least in this model, neuroinflammatory gene expression preceded the response of the peripheral immune system. This has important implications for our present study, as it suggests that continued proinflammatory signaling within the CNS depends on chemoattraction of peripheral immune cells such as monocytes, which is inhibited by the small molecule CVC. Reducing inflammatory signaling in the CNS, which in the case of LPS precedes immune cell infiltration, may contribute to the reduced expression of chemokines. Cell-specific knockout of CCR2 (such as using LysM-driven knockout in peripheral macrophages or CX3CR1-driven knockout in microglia, for example) could help to elucidate the importance of peripheral blockade of CCR2 vs central signaling.

Additionally, recent evidence suggests that, in the developing brain, mice deficient in either CCL2 or CCR2 are protected from alcohol-induced neuroinflammation (including proinflammatory cytokine expression and microglial activation) and neurotoxicity when treated with an acute alcohol exposure at postnatal day 4 [29]. These results agree with the present data, which importantly is from adult mice with chronic alcohol consumption and provide further evidence for the importance of CCL2/CCR2 signaling in alcohol-induced neuroinflammation. Further experiments, including using CCL2 and CCR2 knockout adult mice under chronic alcohol conditions will also be informative.

Interestingly, we observed the most pronounced alterations in the surface activation markers between microglia of alcohol-fed mice and microglia from mice treated with CVC. It is important to note that for flow cytometric analysis for surface marker expression, we used total brain microglia and infiltrating macrophages, whereas other assays (such as histology and biochemical measurements) used more localized populations or tissues for analysis. Therefore, cell surface marker analysis may not fully reflect the micromilieu within subregions of the brain. A possible explanation for the alterations in microglial surface markers is that in the absence of IMs, the remaining microglia are forced to assume a more activated phenotype and respond to the damage induced by alcohol. Interestingly, while we observed surface protein activation markers in microglia, we also observed an activated morphologic phenotype with increased soma size and reduced microglial cell process length. For this analysis, we focused on microglia in the hippocampus, the site of significant peripheral macrophage infiltration (Fig. 2c) and an increase in protein and mRNA expression of proinflammatory cytokines (Fig. 4). Microglial morphology is one indicator of an inflammatory milieu, as these cells change their shape in response to immune activation, and our flow cytometry data suggested that microglia are significantly affected by alcohol and CVC administration (Fig. 5). CVC treatment partially rescued microglial morphology. Compared with alcohol-fed untreated mice, the CVC treatment groups had slightly larger cell bodies but retained the shortened cell process phenotype of alcohol-fed mice. This mixed microglial phenotype, despite the absence of IM infiltration, likely represents the innate response of microglia to alcohol which is characterized by activation and production of proinflammatory cytokines and reactive oxygen species [30–32]. The imaging in Fig. 5 suggests that microglial morphology was altered by chronic alcohol exposure although histologic examination of the complete microglial architecture is technically limited as some microglial processes may be disrupted by the histologic preparation. Additional studies have shown that alcohol consumption may only lead to partial microglia activation [33–35], similar to the findings we present here (Fig. 5a, b). Therefore, we examine the microglial morphologic changes in the context of other measures of a neuroinflammatory milieu including analysis of activation markers and proinflammatory gene expression.

Chronic alcohol exposure induces a complex, multi-organ response with activation of a variety of immune responses and inflammatory expression. Previous research has sought to characterize the gene expression changes caused by chronic alcohol in various parts of the brain. Gene expression analyses in humans [36–39] and rodents [40] reveal alterations in genes related to neurons and neurogenesis, axonal growth, myelin regulation, intracellular signaling, protein trafficking, and other critical cell processes. Interestingly, neuroimmune genes were significantly increased in the frontal cortex of human patients with alcohol use disorder [38], while PET studies using markers of glial density have revealed that alcohol reduces or alters the behavior of CNS glia, likely representing changes in the local neuroimmune milieu [41–43]. Using a targeted screen, we observed alterations in inflammation-related transcripts in the CNS after chronic alcohol in mice and expanded our focus to investigate the hippocampus, cortex, and cerebellum. We observed the upregulation of multiple proinflammatory genes similar to previous descriptions, and some of these were altered by the treatment of CVC to block CCR2/5 signaling. As noted previously, some cytokine expression is increased prior to the infiltration of peripheral immune cells in other neuroinflammation models [28], which may provide an explanation for why CVC treatment only altered the expression of some inflammatory genes in the brain regions investigated.

Our data suggest that infiltrating macrophages specifically target the hippocampus as a site of increased infiltration. The hippocampus is a critical center for learning and memory and has been implicated in the pathology associated with AUD for decades, and this increased peripheral immune cell infiltration may, in part, underline this regional vulnerability. Hippocampal volume loss is associated with alcohol consumption in a dose-dependent manner. In a recently published study, researchers followed individuals for over three decades and show that increasing amounts of alcohol consumption are associated with a greater risk of hippocampal atrophy [3]. Alcohol also influences key processes in memory formation and learning by suppressing long-term potentiation (LTP) of synaptic connections [44–46] and altering proper maturation and maintenance of dendritic spines and synaptic connections within the hippocampus [47, 48].

Microglia play a critical role in the synaptic pruning process [23], and alterations in their normal function have been shown to disrupt proper developmental synaptic pruning [49]. In our study, alcohol consumption led to morphologic changes in microglia and changes in surface marker expression (Fig. 5). Also, the lysosomal protein CD68 was downregulated in the hippocampal microglia. Thus, the effect of alcohol on microglia is significant and may lead to altered microglial phagocytic function. An interesting future direction would be to assess synaptic pruning in this model. There is also evidence that proinflammatory cytokines may have an influence on synapse function, much like complement involvement in the regulation of synapse development. For example, TNFα has been shown to modulate glutamate receptors and decrease synaptic strength [50–52]. Interestingly, while the resident source is likely from the microglia, peripheral macrophages also express TNFα and may be involved in influencing synapses in addition to their possible role promoting neuroinflammation [52, 53]. Additionally, we noted increased cytokine protein expression in the hippocampus (Fig. 4), and cytokines have been implicated in synapse dysregulation in hippocampus neurons in vitro [54]. Alcohol consumption may therefore be inducing microglial activation and changes in cytokine production that could affect the synapse structure function as well as the responsiveness of microglia to CNS immune surveillance and pathogen defense.

Previously, we have used various inhibitors of the proinflammatory NLRP3 inflammasome to show that reduction in inflammation via small molecule inhibitors may affect alcohol consumption and preference. Using therapeutic treatments to inhibit NLRP3, caspase-1, and IL-1β, we found a reduction in ethanol consumption and preference in a two-bottle choice test [18]. Thus, in the treatment of alcohol-related pathology such as alcoholic liver disease, some therapeutics may operate via a mechanism that includes reduced alcohol consumption. Using the two-bottle choice paradigm, we found that CVC treatment did not influence ethanol consumption or preference. Additionally, using the Lieber-DeCarli chronic alcohol consumption model, we observed no difference in alcohol consumption between control and CVC-treated mice. Therefore, we can conclude that the data presented here, which included a reduction of both peripheral macrophage infiltration to the CNS and markers of inflammation within the CNS parenchyma, is likely due to the direct effects of CVC blockade on the CCR2/5 receptors and is not related to a change in the amount of alcohol consumed and/or a preference for alcohol. This is consistent with our previous findings that CVC both prevented and ameliorated alcoholic liver disease in mice [55].

Interestingly, previous behavioral studies by Blednov et al. have shown that CCR2-knockout mice have reduced preference and consumption of ethanol, while CCR5-knockout mice increase their consumption and preference compared with wild-type mice [5]. CVC acts as a dual inhibitor blocking both the CCR2 and CCR5 receptors. Therefore, our observation of no change in consumption or preference in CVC-treated mice may be explained by the previous observations that CCR2 and CCR5 genetic deficiencies have opposite effects on alcohol use or preference. Using a dual inhibitor, we may have negated any effect on ethanol-seeking behavior and thus isolated a direct biologic effect of the CVC molecule on the underlying biologic pathology associated with alcohol use rather than influencing behavior and ethanol exposure.

Inflammatory processes in disease states generally occur as an evolutionary beneficial host response. However, chronic dysregulated inflammation often contributes to organ pathology and disease and is a key feature of chronic alcohol exposure. Infiltrating macrophages to the CNS in the setting of chronic alcohol may be protective or detrimental to the overall CNS milieu. Whereas we have previously shown that CVC reduces features of alcoholic liver disease, the hallmark pathologic features of chronic alcohol on the CNS are continuing to be defined, especially within the realm of inflammation, and this present study stimulates many interesting areas for future research including on synapse structures and functions, functional connections between glia and neurons, viability of neurons and glial cells, integrity of the blood-brain barrier, global neuronal network connections, and more.

## Conclusion

In conclusion, we report here that chronic alcohol induces region-specific infiltration of IMs into the CNS and that inhibition of CCR2/5 signaling using the small molecule inhibitor CVC abrogated the infiltration of macrophages. Chronic alcohol also induced cytokine expression and microglial activation that was partially normalized by CCR2/5 inhibition. These data provide critical insights into the role of CCR2/5 signaling, IMs, and microglia in alcohol-induced neuroinflammation.

## Supplementary information

**Additional file 1: Figure S1.** Ethanol preference and consumption is unaffected by CVC treatment. Mice were housed singly and received daily injection of CVC. Two-bottle choice of water or alcohol in drinking water at increasing concentrations (3, 6, 9, and 12%) for 4 days each was provided ad libitum. Consumption was measured, and mice were provided freshwater and alcohol daily. A) The amount of ethanol consumed at each concentration was normalized to mouse body weight (BW). B) Alcohol preference was determined as a ratio of the volume of alcohol consumed to the total liquid volume consumed per day. C) Total fluid intake (water plus alcohol) was measured and normalized to the respective body weight of each mouse. Data are mean +/- SEM, *n* = 8 mice/group. Two-way ANOVA with Bonferroni correction results are described in the text.

## Data Availability

All data generated or analyzed during this study is contained within the manuscript.

## Abbreviations

AUD: Alcohol use disorder
CNS: Central nervous system
CVC: Cenicriviroc
IMs: Infiltrating macrophages
DG: Dentate gyrus

## References

[1] National Institute on Alcohol Abuse and Alcoholism. 2017. https://www.niaaa.nih.gov/alcohol-health/overview-alcohol-consumption/alcohol-facts-and-statistics.

[2] Crews FT (2004). Alcohol-induced neurodegeneration: when, where and why?. Alcohol Clin Exp Res.

[3] Topiwala A (2017). Moderate alcohol consumption as risk factor for adverse brain outcomes and cognitive decline: longitudinal cohort study. BMJ.

[4] Szabo G, Lippai D (2014). Converging actions of alcohol on liver and brain immune signaling. Int Rev Neurobiol.

[5] Blednov YA (2005). Perturbation of chemokine networks by gene deletion alters the reinforcing actions of ethanol. Behav Brain Res.

[6] Blednov YA (2012). Neuroimmune regulation of alcohol consumption: behavioral validation of genes obtained from genomic studies. Addict Biol.

[7] Kelley KW, Dantzer R (2011). Alcoholism and inflammation: neuroimmunology of behavioral and mood disorders. Brain Behav Immun.

[8] He J, Crews FT (2008). Increased MCP-1 and microglia in various regions of the human alcoholic brain. Exp Neurol.

[9] Vetreno RP, Qin L, Crews FT (2013). Increased receptor for advanced glycation end product expression in the human alcoholic prefrontal cortex is linked to adolescent drinking. Neurobiol Dis.

[10] Miro-Mur F (2016). Immature monocytes recruited to the ischemic mouse brain differentiate into macrophages with features of alternative activation. Brain Behav Immun.

[11] Rubio-Araiz A (2017). Disruption of blood-brain barrier integrity in postmortem alcoholic brain: preclinical evidence of TLR4 involvement from a binge-like drinking model. Addict Biol.

[12] Lippai D, Bala S, Csak T, Kurt-Jones EA, Szabo G (2013). Chronic alcohol-induced microRNA-155 contributes to neuroinflammation in a TLR4-dependent manner in mice. PLoS One.

[13] Lippai D (2013). Alcohol-induced IL-1beta in the brain is mediated by NLRP3/ASC inflammasome activation that amplifies neuroinflammation. J Leukoc Biol.

[14] Marques RE, Guabiraba R, Russo RC, Teixeira MM (2013). Targeting CCL5 in inflammation. Expert Opin Ther Targets.

[15] Szabo G, Petrasek J (2015). Inflammasome activation and function in liver disease. Nat Rev Gastroenterol Hepatol.

[16] Lefebvre E (2016). Antifibrotic effects of the dual CCR2/CCR5 antagonist cenicriviroc in animal models of liver and kidney fibrosis. PLoS One.

[17] Iracheta-Vellve A (2015). Inhibition of sterile danger signals, uric acid and ATP, prevents inflammasome activation and protects from alcoholic steatohepatitis in mice. J Hepatol.

[18] Lowe PP (2020). Inhibition of the inflammasome signaling cascade reduces alcohol consumption in female but not male mice. Alcohol Clin Exp Res.

[19] Monell Mouse Taste Phenotyping Project. *Construction of graduated drinking tubes*, <http://www.monell.org/MMTPP/Drinking%20tubes.htm> (1999).

[20] Wilkin RJ, Lalor PF, Parker R, Newsome PN (2016). Murine models of acute alcoholic hepatitis and their relevance to human disease. Am J Pathol.

[21] Zimmermann HW, Tacke F (2011). Modification of chemokine pathways and immune cell infiltration as a novel therapeutic approach in liver inflammation and fibrosis. Inflamm Allergy Drug Targets.

[22] Alfonso-Loeches S, Urena-Peralta J, Morillo-Bargues MJ, Gomez-Pinedo U, Guerri C (2016). Ethanol-induced TLR4/NLRP3 neuroinflammatory response in microglial cells promotes leukocyte infiltration across the BBB. Neurochem Res.

[23] Schafer DP (2012). Microglia sculpt postnatal neural circuits in an activity and complement-dependent manner. Neuron.

[24] Chistiakov DA, Killingsworth MC, Myasoedova VA, Orekhov AN, Bobryshev YV (2017). CD68/macrosialin: not just a histochemical marker. Lab Invest.

[25] Greter M, Lelios I, Croxford AL (2015). Microglia versus myeloid cell nomenclature during brain inflammation. Front Immunol.

[26] Zou J, Crews FT (2012). Inflammasome-IL-1β signaling mediates ethanol inhibition of hippocampal neurogenesis. Front Neurosci.

[27] Zou J, Crews F (2010). Induction of innate immune gene expression cascades in brain slice cultures by ethanol: key role of NF-κB and proinflammatory cytokines. Alcohol Clin Exp Res.

[28] Rankine EL, Hughes PM, Botham MS, Perry VH, Felton LM (2006). Brain cytokine synthesis induced by an intraparenchymal injection of LPS is reduced in MCP-1-deficient mice prior to leucocyte recruitment. Eur J Neurosci.

[29] Zhang K, Wang H, Xu M, Frank JA, Luo J (2018). Role of MCP-1 and CCR2 in ethanol-induced neuroinflammation and neurodegeneration in the developing brain. J Neuroinflammation.

[30] Fernandez-Lizarbe S, Montesinos J, Guerri C (2013). Ethanol induces TLR4/TLR2 association, triggering an inflammatory response in microglial cells. J Neurochem.

[31] Boyadjieva NI, Sarkar DK (2013). Microglia play a role in ethanol-induced oxidative stress and apoptosis in developing hypothalamic neurons. Alcohol Clin Exp Res.

[32] Gofman L, Cenna JM, Potula R (2014). P2X4 receptor regulates alcohol-induced responses in microglia. J Neuroimmune Pharmacol.

[33] Cruz C, Meireles M, Silva SM (2017). Chronic ethanol intake induces partial microglial activation that is not reversed by long-term ethanol withdrawal in the rat hippocampal formation. Neurotoxicology.

[34] Marshall SA (2013). Microglial activation is not equivalent to neuroinflammation in alcohol-induced neurodegeneration: the importance of microglia phenotype. Neurobiol Dis.

[35] Qin L (2008). Increased systemic and brain cytokine production and neuroinflammation by endotoxin following ethanol treatment. J Neuroinflammation.

[36] Mayfield RD (2002). Patterns of gene expression are altered in the frontal and motor cortices of human alcoholics. J Neurochem.

[37] Lewohl JM (2000). Gene expression in human alcoholism: microarray analysis of frontal cortex. Alcohol Clin Exp Res.

[38] Liu J (2006). Patterns of gene expression in the frontal cortex discriminate alcoholic from nonalcoholic individuals. Neuropsychopharmacology.

[39] Flatscher-Bader T (2005). Alcohol-responsive genes in the frontal cortex and nucleus accumbens of human alcoholics. J Neurochem.

[40] McClintick JN (2018). Gene expression changes in the ventral hippocampus and medial prefrontal cortex of adolescent alcohol-preferring (P) rats following binge-like alcohol drinking. Alcohol.

[41] Kalk NJ (2017). Decreased hippocampal translocator protein (18 kDa) expression in alcohol dependence: a [^11^C]PBR28 PET study. Transl Psychiatry.

[42] Kim SW (2018). Influence of alcoholism and cholesterol on TSPO binding in brain: PET [^11^C]PBR28 studies in humans and rodents. Neuropsychopharmacology.

[43] Hillmer AT (2017). In vivo imaging of translocator protein, a marker of activated microglia, in alcohol dependence. Mol Psychiatry.

[44] Blitzer RD, Gil O, Landau EM (1990). Long-term potentiation in rat hippocampus is inhibited by low concentrations of ethanol. Brain research.

[45] Schummers J, Bentz S, Browning MD (1997). Ethanol’s inhibition of LTP may not be mediated solely via direct effects on the NMDA receptor. Alcohol Clin Exp Res.

[46] Pyapali GK, Turner DA, Wilson WA, Swartzwelder HS (1999). Age and dose-dependent effects of ethanol on the induction of hippocampal long-term potentiation. Alcohol.

[47] Risher ML (2015). Adolescent intermittent alcohol exposure: persistence of structural and functional hippocampal abnormalities into adulthood. Alcohol Clin Exp Res.

[48] Risher ML (2015). Adolescent intermittent alcohol exposure: dysregulation of thrombospondins and synapse formation are associated with decreased neuronal density in the adult hippocampus. Alcohol Clin Exp Res.

[49] Kim HJ (2017). Deficient autophagy in microglia impairs synaptic pruning and causes social behavioral defects. Mol Psychiatry.

[50] Lewitus GM (2016). Microglial TNF-α suppresses cocaine-induced plasticity and behavioral sensitization. Neuron.

[51] Stellwagen D, Malenka RC (2006). Synaptic scaling mediated by glial TNF-α. Nature.

[52] Werneburg S, Feinberg PA, Johnson KM, Schafer DP (2017). A microglia-cytokine axis to modulate synaptic connectivity and function. Curr Opin Neurobiol.

[53] Zhang Y (2014). An RNA-sequencing transcriptome and splicing database of glia, neurons, and vascular cells of the cerebral cortex. J Neurosci.

[54] Lim SH (2013). Neuronal synapse formation induced by microglia and interleukin 10. PLoS One.

[55] Ambade A (2019). Pharmacological inhibition of CCR2/5 signaling prevents and reverses alcohol-induced liver damage, steatosis, and inflammation in mice. Hepatology.

