# Supplementary material for: Chronic alcohol-induced neuroinflammation involves CCR2/5-dependent peripheral macrophage infiltration and microglia alterations
Source: J Neuroinflammation. 2020 Oct 9;17:296. doi: 10.1186/s12974-020-01972-5 (PMC7547498; doi:10.1186/s12974-020-01972-5)
Supplement: Supplementary file 1 [file 12974_2020_1972_MOESM1_ESM.docx]

Figure S1

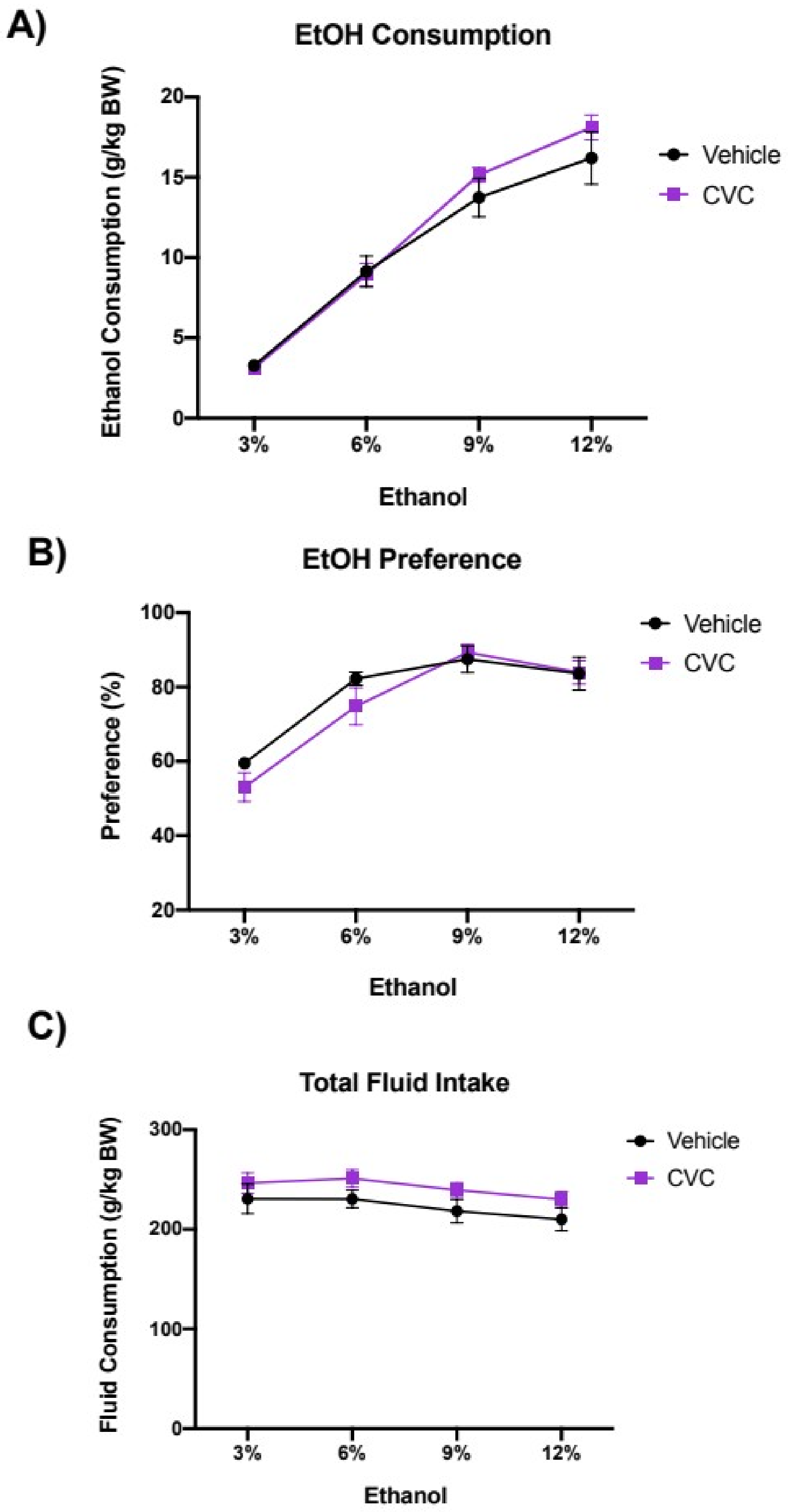
A)
EtOH Consumption
Ethanol Consumption (g/kg BW)
20
15
10
5
0
3%
6%
9%
12%
Ethanol
Vehicle
CVC
B)
EtOH Preference
Preference (%)
100
80
60
40
20
3%
6%
9%
12%
Ethanol
Vehicle
CVC
C)
Total Fluid Intake
Fluid Consumption (g/kg BW)
300
200
100
0
3%
6%
9%
12%
Ethanol
Vehicle
CVC
